# A Novel Pipeline Leak Detection Technique Based on Acoustic Emission Features and Two-Sample Kolmogorov–Smirnov Test

**DOI:** 10.3390/s21248247

**Published:** 2021-12-10

**Authors:** Akhand Rai, Zahoor Ahmad, Md Junayed Hasan, Jong-Myon Kim

**Affiliations:** 1 School of Engineering and Applied Science, Ahmedabad University, Ahmedabad 380009, Gujarat, India; akhand.rai@ahduni.edu.in; 2 Department of Electrical, Electronics and Computer Engineering, University of Ulsan, Ulsan 44610, Korea; zahooruou@mail.ulsan.ac.kr (Z.A.); junhasan@mail.ulsan.ac.kr (M.J.H.); 3 PD Technology Co., Ulsan 44610, Korea

**Keywords:** pipeline, leak detection, acoustic emission, Kolmogorov–Smirnov test

## Abstract

Pipeline leakage remains a challenge in various industries. Acoustic emission (AE) technology has recently shown great potential for leak diagnosis. Many AE features, such as root mean square (RMS), peak value, standard deviation, mean value, and entropy, have been suggested to detect leaks. However, background noise in AE signals makes these features ineffective. The present paper proposes a pipeline leak detection technique based on acoustic emission event (AEE) features and a Kolmogorov–Smirnov (KS) test. The AEE features, namely, peak amplitude, energy, rise-time, decay time, and counts, are inherent properties of AE signals and therefore more suitable for recognizing leak attributes. Surprisingly, the AEE features have received negligible attention. According to the proposed technique, the AEE features are first extracted from the AE signals. For this purpose, a sliding window was used with an adaptive threshold so that the properties of both burst- and continuous-type emissions can be retained. The AEE features form distribution that change its shape when the pipeline condition changes from normal to leakage. The AEE feature distributions for leak and healthy conditions were discriminated using the two-sample KS test, and a pipeline leak indicator (PLI) was obtained. The experimental results demonstrate that the developed PLI accurately distinguishes the leak and no-leak conditions without any prior leak information and it performs better than the traditional features such as mean, variance, RMS, and kurtosis.

## 1. Introduction

Leaks in pipelines create fluid supply system malfunctions potentially leading to discharge of hazardous materials into the environment, undue maintenance expenses, increased repair costs, system downtime losses, and severe accidents. Therefore, a continuous inspection of the pipeline condition is important to detect leaks. Over the past few decades, numerous external and internal monitoring methods have been proposed for pipeline leak detection, including negative pressure wave (NPW) techniques [1], accelerometer-based techniques [2], acoustic emission (AE) technology [3], time-domain reflectometry [4], distributed temperature sensing systems [5], ultrasonic technology [6], and magnetic flux leakage techniques [7]. AE technologies have gained significant popularity because of its rapid leak detection capability, high sensitivity, real-time response, and ease of retrofitting [8]. A significant amount of research associated with AE expertise primarily uses feature extraction and pattern recognition techniques to build the leak detection models [9,10,11,12]. Xiao et al. [13] employed wavelet features and a support vector machine (SVM) for classifying the leak and non-leak states. Wang et al. [14] extracted frequency–width features from the time-domain pipeline signals and used them to train a support vector data description (SVDD) model to detect leaks. Zadkarami et al. [15] used a multi-layer perceptron neural network (MLPNN) and Dempster–Shafer classifier fusion technique for learning the leak patterns represented by statistical and wavelet-based features. Li et al. [16] suggested a leak detection approach based on kernel principal component analysis (KPCA) and SVM; the KPCA was employed to extract optimal leak features and achieve better SVM classification. Sun et al. [2] used envelope spectrum entropy features obtained by local mean decomposition (LMD) of the AE signals to train SVM and recognize leaks. Sun et al. [17] further applied LMD and WT to extract RMS entropy features that were then used for building an SVM-leak detection model. Cui et al. [18] used empirical mode decomposition (EMD) to process the non-stationary pipeline signals and detect leaks in CO2 gas pipelines. Xu et al. [19] used the wavelet packet transform (WT) and time-domain features, such as mean value, peak value, RMS value, standard deviation, peak index, pulse index, waveform index, and root amplitude, in combination with Fuzzy SVM for identifying leaks. Although these studies were appropriate for leak diagnosis, they suffered from several shortcomings. First, failure data associated with a pipeline leak are required to train the supervised learning techniques such as SVM and MLPNN.

In industrial applications, it is impractical to attain the leak data because the damaged pipeline is immediately replaced to prevent any harmful repercussions. Second, the pattern recognition methods require a large amount of data for training. Insufficient training data produces inaccurate results when classifying the leak conditions from the normal conditions. Third, the earlier feature extraction techniques may fail to represent the leak signals accurately. AE signals are heavily affected by attenuation and surrounding noise, causing unexpected changes in the AE waveform distribution. The time-domain features are sensitive to such variations, creating a false impression of the pipeline condition. The frequency domain features are corrupted by undesirable frequency components generated because of external equipment such as pumps and motors. The time–frequency domain techniques, such as WT, EMD, and LMD, are dependent on the selection of optimal wavelet bases and frequency modes for extraction of reliable fault features. Additionally, the time–frequency decomposition of large AE signals is time-consuming. To overcome these shortcomings, this paper proposes a novel approach using AE-event features and a two-sample Kolmogorov–Smirnov (KS) test for pipeline leak detection. 

The commonly used AE-event (AEE) features are peak amplitude, energy, rise time, decay time, and counts. The AE-event features primarily focus on the information in the events or bursts formed in the AE signals because of faults. Consequently, they are independent of the entire signal distribution and less sensitive to outliers. These features have demonstrated excellent capabilities to address AE signals from different applications such as concrete structures and composites [20,21,22,23]. Surprisingly, the AEE features have been rarely used to detect leaks in pipelines. To address this research gap, the current paper attempts to explore the potential of AEE features for recognizing pipeline leaks. Moreover, the two-sample KS test was used to model the information in the AEE feature data and recognize pipeline leaks. The two-sample KS test is a hypothesis testing technique used to discriminate between the cumulative distribution functions of two data samples [24]. As such, it can be used to distinguish between the AEE feature data distributions of normal and leak states in order to reveal the pipeline condition. The two-sample KS-test was used successfully for fault diagnosis of bearings, pumps, and gears [25,26,27]. The KS test offers the advantage that it does not need any prior leak information or a large amount of training data. 

The rest of this paper is organized as follows. Section 2 describes the theoretical background behind AEE features and the two-sample KS test. Section 3 provides the outline of the proposed approach. The details of the experimental setup are given in Section 4. Section 5 discusses the experimental results. Section 6 concludes the work. 

## 2. Technical Background

### 2.1. Acoustic Emission Event (AEE) Features

**Figure 1:**
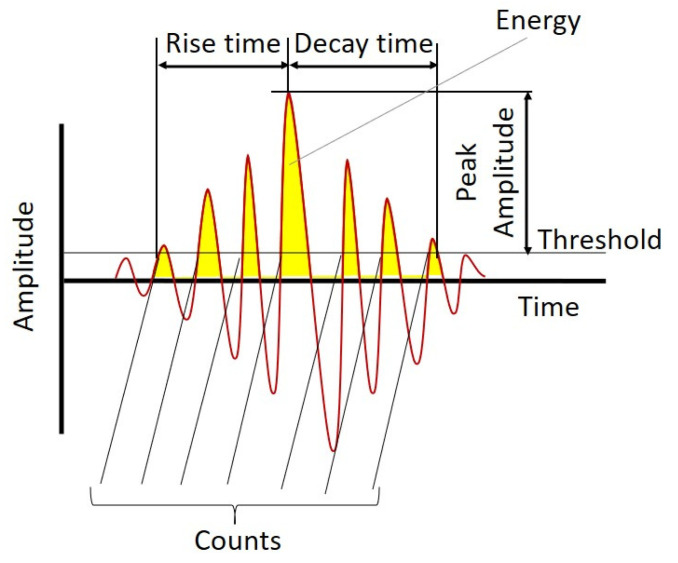
AEE features.

Leaks in a pipelines create stress waves that are transmitted through the pipe walls and recorded through AE sensors installed on the pipeline. These stress waves produce transients commonly referred to as AE events or hits. Figure 1 depicts an overview of AEE and the extracted features. A threshold is set above the level of continuous background noise to declare an event and avoid false triggers. The AEE features can be defined as follows [20,21,22,23]:

*Peak amplitude:* Maximum amplitude of AEE;

*Energy:* Area under the AEE waveform;

*Rise time:* Time taken by AEE to reach the maximum value beginning from the moment of the first threshold crossing;

*Decay time:* Time taken by the AEE to decay from the maximum value to the threshold level;

*Counts:* Number of times AEE crosses the threshold. 

### 2.2. Two Sample KS Test

The two-sample KS test distinguishes between two datasets by comparing their empirical cumulative distribution functions (ECDFs) [25,26,27]. If the two samples have identical ECDFs, they are drawn from the same population; otherwise, they belong to different populations. Mathematically, the hypothesis can be formulated as follows:

**Null** **Hypothesis** **0** **(H0).** *F(x) = G(x).*

**Alternative** **Hypothesis** **1** **(H1).** *F(x)≠ G(x).*

where *F*(*x*) and *G*(*x*) denote the ECDFs of two data distributions tested for similarity. The ECDFs *F*(*x*) and *G*(*x*) are computed as follows:

(1) $$\begin{array}{l} F\left(X^{1}\right)=P\left(X_{i}^{1}\leq x\right)=k_{1}/N_{1}\;-\infty <x<\infty \\ G\left(X^{2}\right)=P\left(X_{i}^{2}\leq x\right)=k_{2}/N_{2} \end{array}$$

where *X*^1^ and *X*^2^ represent the observations from two datasets, *P* (*X_i_* ≤ *x*) denotes the probability of observations less than or equal to *X_i_*, *k*_1_ and *k*_2_ are the number of observations less than or equal to *X_i_*^1^ and *X_i_*^2^, respectively, and *N*_1_ and *N*_2_ are the total number of observations in the two samples. 

To validate the above null hypothesis, a distance measure, *d*-statistic (*d*-stat), is calculated. If the *d*-stat value exceeds the critical value *±dα* at significance level *α*, then the null hypothesis is rejected. It is defined as the maximum of absolute difference between two ECDFs, *F*(*x*) and *G*(*x*). Mathematically, d-stat can be expressed as:

(2) $$d-stat=\max\left\{\left|F\left(X_{i}^{1}\right)-G(X_{i}^{2})\right|\right\}$$

where the ECDFs, *F*(*x*) and *G*(*x*), are computed for observations *X_i_*^1^ and *X_i_*^2^, respectively.

It is feasible to exploit the *d*-stat as the pipeline health indicator. The current datasets acquired from the pipeline can be tested for similarity with those taken from healthy pipeline conditions. When the test and healthy datasets have similar ECDFs, a low value of *d*-stat is obtained. As soon as a leak occurs, the ECDF of the test dataset becomes dissimilar to that of the healthy dataset and an increase in the *d*-stat value is observed.

## 3. Proposed Approach

**Figure 2:**
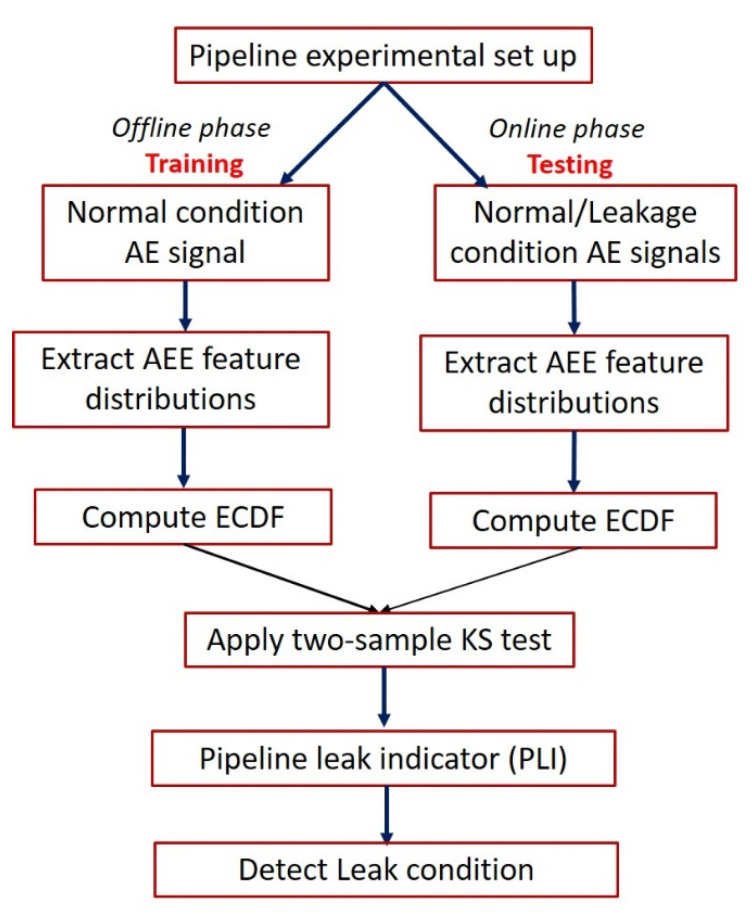
Layout of the proposed approach.

**Figure 3:**
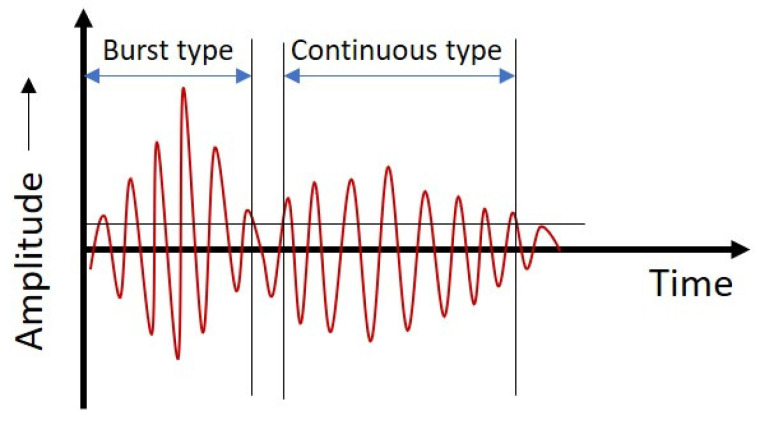
Burst- and continuous-type AEEs.

Figure 2 depicts the layout of the proposed approach. The proposed leak detection procedure is implemented in the following steps:

1. The AE signals are acquired from the pipeline to be monitored;
2. The AEE features described in Section 2 (A) are extracted from AE signals. An AE signal contains multiple events occurring because of leakage. These AEEs can be either continuous or burst type, as shown in Figure 3. The burst-type AE events are easily distinguishable from the background noise as compared with continuous AE events. As such, proper setting of the threshold is essential to extract useful features from both burst and continuous-type AEEs. Therefore, for preventing information loss, a sliding window of arbitrary length ‘s’ is taken to possibly cover every event, and the threshold is computed for each sliding window rather than setting a common threshold for the entire AE signal. Thus, the AEE threshold adapts itself to the attributes of a particular window. References [20,21,22,23] suggest that the peak value is directly related to the AEE characteristics. Consequently, the adaptive threshold value is taken equal to a certain percentage ‘p’ of the peak amplitude in a sliding window;
3. The values of the AEE features for different windows produce distributions that change their shape and size as the pipeline state shifts from healthy to leakage. This variation is detected by applying the two-sample KS test and computing the d-stat value using Equation (2). Thus, the d-stat value is used as the pipeline leak indicator (PLI). An increase in the d-stat value will possibly signify the commencement of a leak in pipeline. For better interpretation of the analysis, the Equation (2) is rewritten as:

(3) $$PLI=\max\left\{\left|F\left(X_{i}^{N}\right)-G(X_{i}^{L})\right|\right\}$$

where *X_i_^N^* and *X_i_^L^* represent the observations corresponding to the normal and leak conditions, respectively; *F*(*X_i_*^1^) and *G*(*X_i_*^2^) represent the ECDFs computed for observations *X_i_^N^* and *X_i_^L^*, respectively.

## 4. Experimental Setup

**Figure 4:**
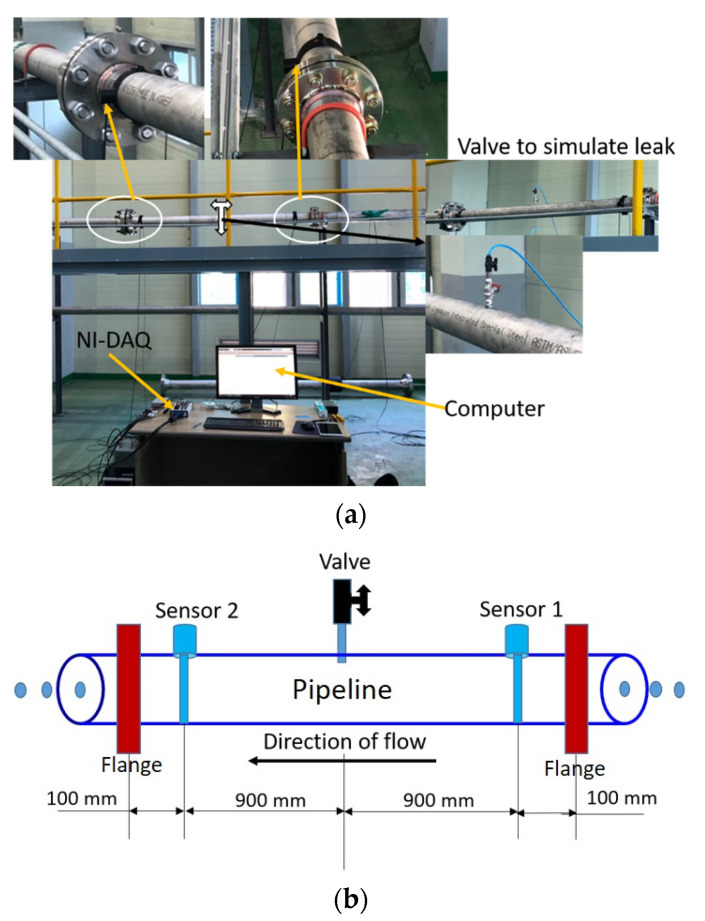
Experimental setup used for simulating leaks in the pipelines. (**a**) Original photographs. (**b**) Schematic diagram.

The pipeline AE signals used to validate the proposed technique are gathered from the experimental set up shown in Figure 4a,b. For complete details on the experimental setup, readers are advised to refer to our previous publication [28]. However, a brief overview is given here for a quick reference to the readers. Figure 4a portrays the original photographs of the test setup and Figure 4b depicts the corresponding schematic diagram. The experimental arrangement primarily consists of a pipeline carrying water and necessary data collection accessories such as sensors and a computer. A valve is installed on the pipeline to simulate leaks. The location of the simulated leaks is depicted in Figure 4b. The pipeline material is Stainless Steel 304. The outer diameter and thickness of the pipeline are 114 and 6 mm, respectively. The AE sensors attached to the pipeline via glue gels and mounting tapes are shown in Figure 4a. The AE sensor type is R15I-AST manufactured by MISTRAS Group, Inc. A NI-9223 National Instruments Data Acquisition (NI-DAQ) system along with a programmed computer are used to capture the pipeline condition data. The sampling frequency was set to 1 MHz. Pencil lead break (PLB) tests were conducted to confirm the responsiveness of the sensors to the applied load. Initially, the valve remains closed and the pipeline runs under the normal condition. The pressure in the pipeline is maintained at 7 bar and the data are collected for 2 min. Afterwards, a leak of size 0.3 mm was introduced to the pipeline by opening the valve, and the data were acquired for the next 2 min. The valve was closed again and the flow in the pipeline was steadied. The test was then repeated at 13 bar for the same leak size of 0.3 mm. In a similar manner, the pipeline data were gathered for leaks sizes of 0.5 and 1 mm at 7 and 13 bar, respectively. Thus, for each test, i.e., a particular leak size and pressure, 240 signal samples were collected. Of these 240 samples, 60 samples from the normal condition, and 60 samples from the leak condition were considered for further analysis. 

## 5. Results and Discussion

**Figure 5:**
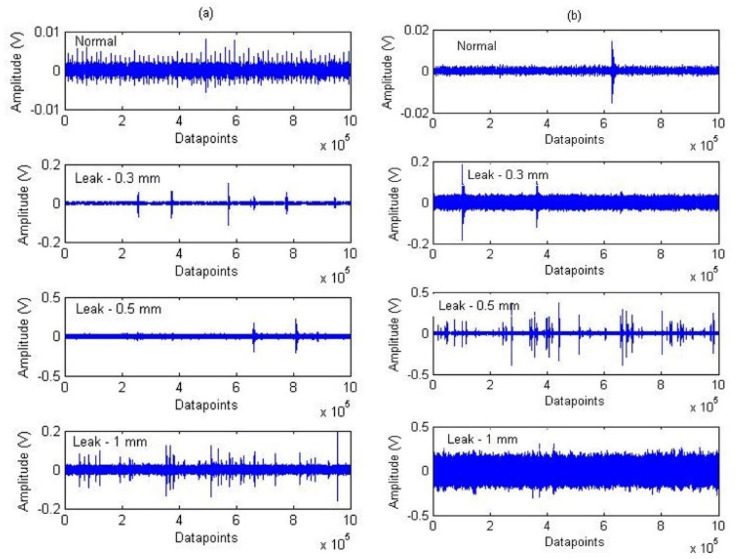
AE signals for normal and leak conditions of the pipeline at pressures of (**a**) 7 and (**b**) 13 bar.

The experimental results of the proposed approach are discussed in this section. Figure 5 shows a plot of the AEE features calculated from a signal sample acquired under normal and leak conditions corresponding to a 0.3 mm leak size and 7 bar pressure. To extract the AEE features, a 0.1 s signal of length equal to 100,000 data points was considered. The length of the sliding window ‘s’ and adaptive threshold value ‘p’ are taken as 1000 data points and 10%, respectively. 

**Figure 6:**
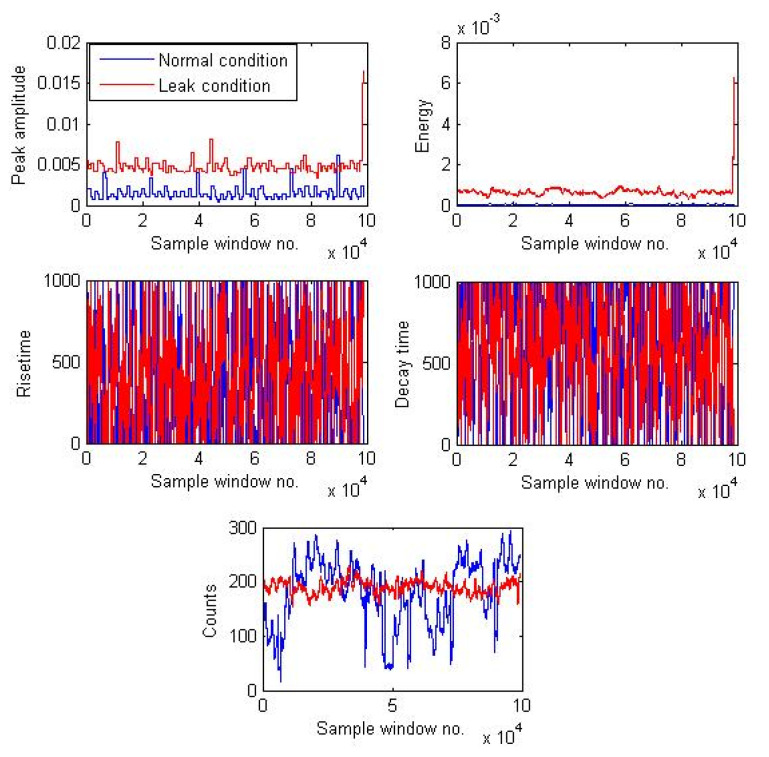
AEE features calculated for signal samples corresponding to normal and a 0.3-mm size leak under 7 bar pressure conditions.

Figure 6 shows a plot of the AEE features extracted from two different signal samples collected under 7 bar pressure, one corresponding to the normal condition and another corresponding to the leak size of 0.3 mm. 

**Figure 7:**
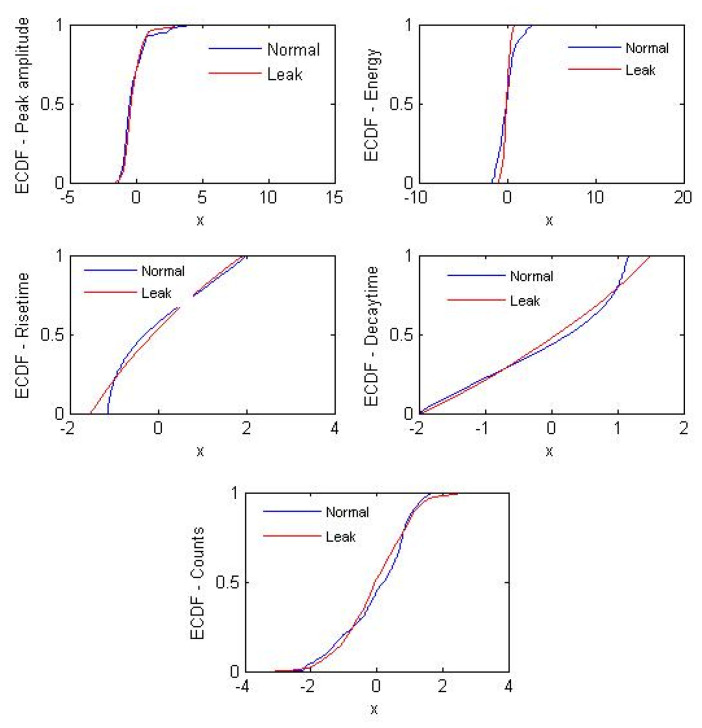
ECDF plots for the different AEE features calculated for signal samples corresponding to a normal and 0.3-mm leak size acquired under 7 bar pressure.

Figure 7 shows the cumulative probability distribution plots corresponding to the AEE features shown in Figure 6. The cumulative distribution of AEE features for the normal and leak conditions have a noticeable difference. This is because of the fact that as the pipeline condition changes from normal to leakage, the AE signal attributes also change due to the generation of elastic waves by the leaks. This behavior was exploited to distinguish between the normal and leak conditions by computing the PLI defined by Equation (3). 

**Figure 8:**
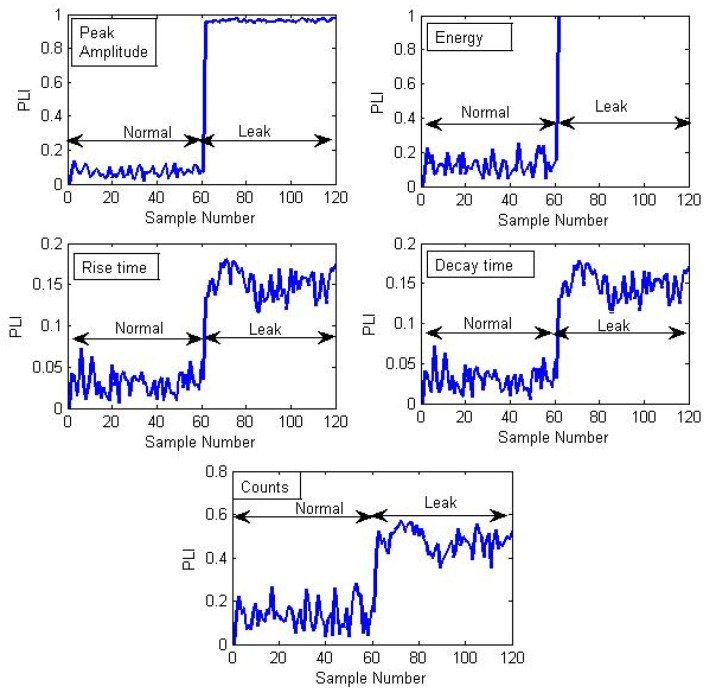
PLI plots corresponding to different AEE features for the experimental test conducted for a 0.3-mm leak size and 7 bar pressure.

Figure 8 depicts the PLIs obtained for the experimental tests conducted for the leak of size 0.3 mm under 7 bar pressure and obtained by substituting different AEE feature distributions into the two-sample KS test. As shown in Figure 8, the PLIs clearly distinguish between the normal and leak conditions. Moreover, the PLIs associated with each AEE feature change by a different amount when the pipeline condition switches from normal to leakage. As soon as the leak occurs, the PLIs show a significant jump indicating the separation in ECDFs of AEE features associated with normal and leak signals. This may be beneficial in making sure that the leak has occurred because the PLI should vary for each of the AEE features. 

**Figure 9:**
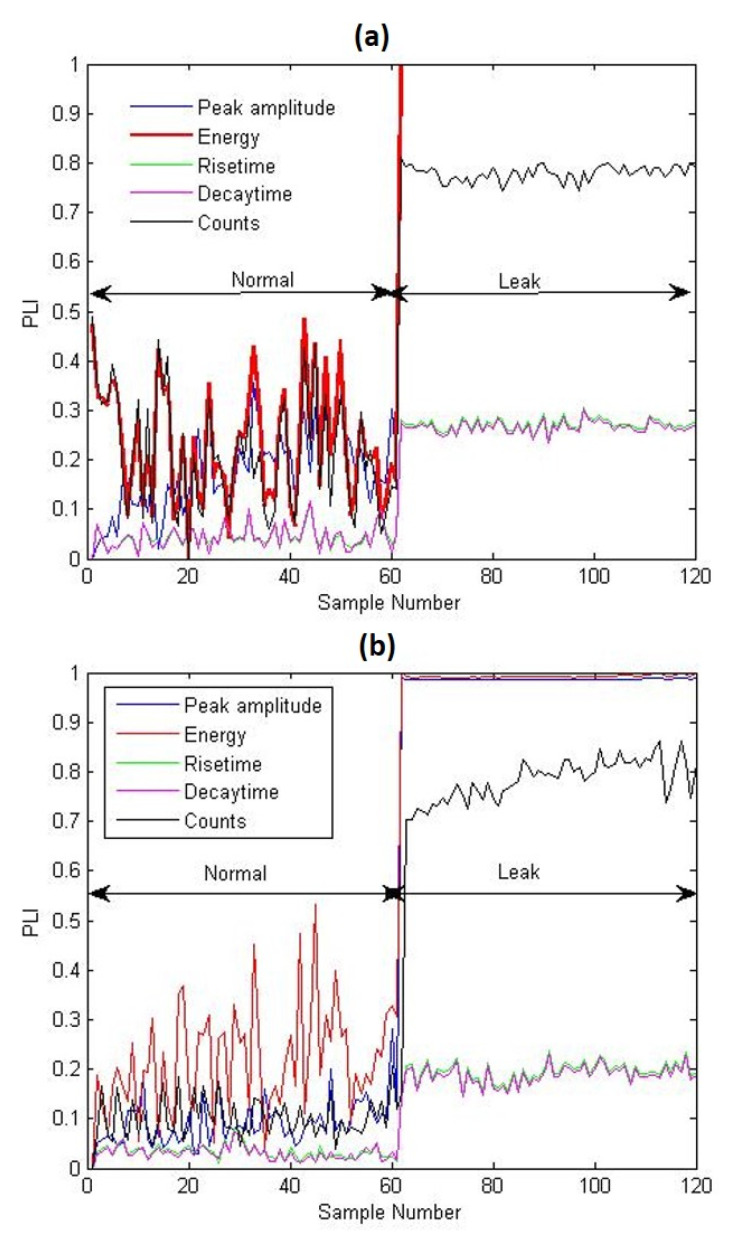
PLI plots corresponding to different AEE features for the experimental tests conducted for leaks of sizes (**a**) 0.5 and (**b**) 1 mm.

When the PLI changes only for a single feature, outliers may be present rather than a leakage. Similar positive analysis results were obtained for 0.5- and 1-mm size leak tests, as shown in Figure 9a,b. Therefore, the PLIs are capable of detecting leaks of varying sizes without prior leak data. 

**Figure 10:**
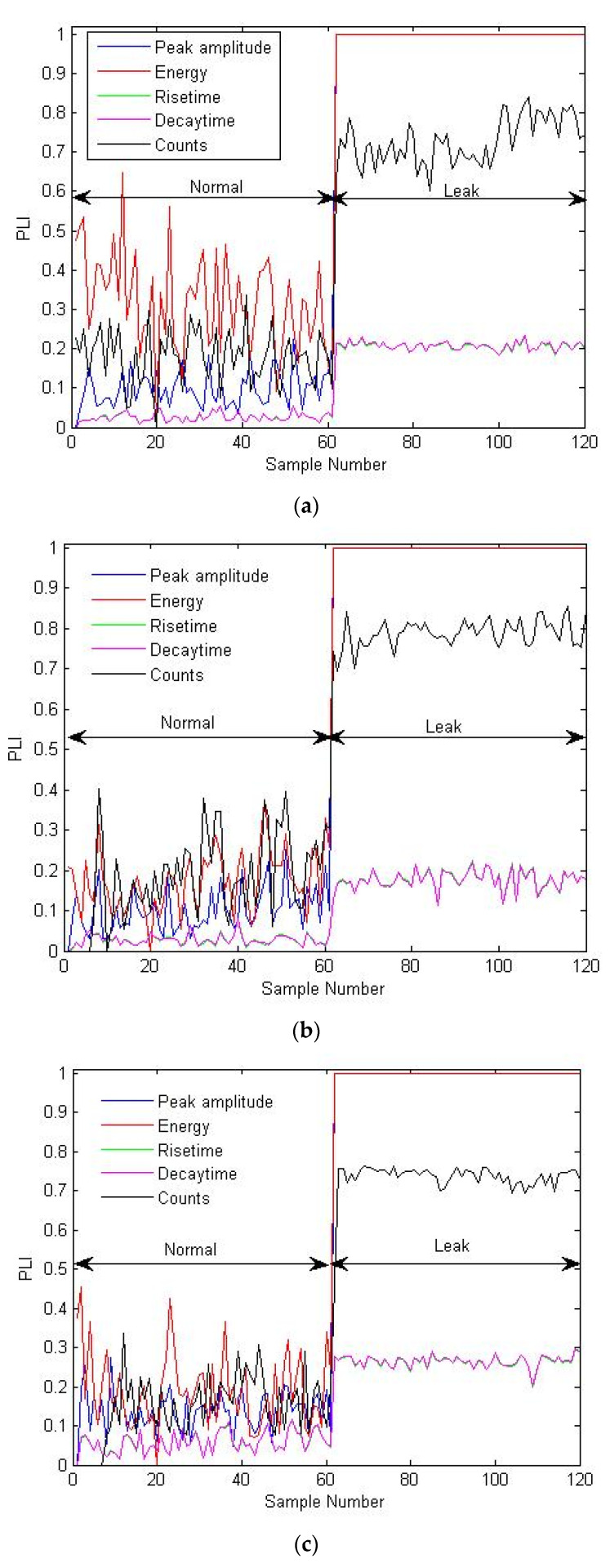
PLI plots corresponding to different AEE features for the experimental tests carried out at 13 bar pressure for leaks of sizes (**a**) 0.3, (**b**) 0.5, and (**c**) 1 mm.

The proposed approach was again tested on the experimental tests conducted at 13 bar pressure and the corresponding PLI plots are shown in Figure 10a–c. The PLIs successfully identified the leak conditions by reporting a significant jump on the occurrence of leaks, even if the pressure condition changed to 13 bar. In summary, the PLI derived using the proposed approach exhibits good performance in detecting the leak conditions, irrespective of the leak size and pressure conditions, and they do not require any preceding leak information for their construction. In addition, the PLIs lie between 0 and 1, which is yet another advantage because it helps in establishing definite thresholds for leak situations. 

**Figure 11:**
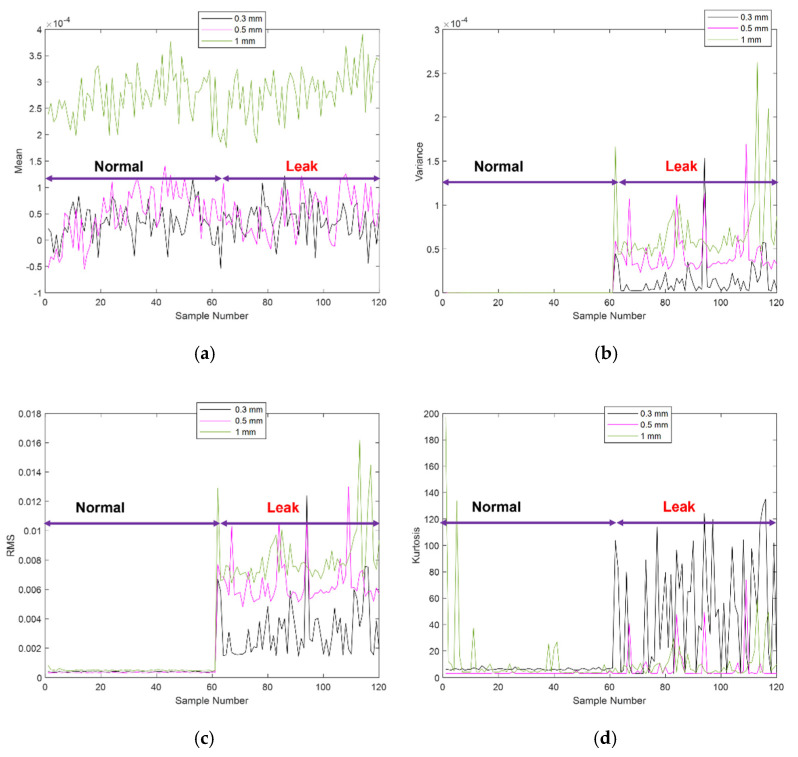
Traditional features extracted for the experimental tests at 7 bar pressure and leaks of sizes, 0.3, 0.5, and 1 mm (**a**) mean, (**b**) variance, (**c**) RMS, (**d**) kurtosis.

To further validate the effectiveness of the proposed approach, the derived PLIs were compared with four commonly used features: mean, variance, RMS, and kurtosis. The plots of these traditional features for 7 bar pressure and leak sizes of 0.3, 0.5, and 1 mm are shown in Figure 11. The mean and kurtosis features fluctuate at nearly the same level and fail to separate the normal and different leak conditions. The variance feature works for 0.5- and 1-mm leaks; however, it was inaccurate for a smaller leak of 0.3 mm. Only the RMS feature was successful in recognizing the leak conditions. However, the PLI features shows a greater sensitivity towards the leak conditions than the RMS feature. 

To confirm this, a new index, the leak sensitivity, was defined, which is measured as the average deviation in PLI values of the leak conditions with respect to the normal condition. The leak sensitivity is calculated by Equation (4).

(4) $$Leak\;senstivity=\frac{\sum_{1}^{T}\left(PLI_{LEAK}-PLI_{NORMAL}\right)}{T}$$

where *PLI_LEAK_* and *PLI_NORMAL_* denote the leak indicator values corresponding to the leak and normal conditions, and T is the number of leak samples.

The leak sensitivity values calculated for the different PLIs corresponding to 7 bar pressure and 0.3 mm leak are Peak amplitude: 0.88, Energy: 0.8, Rise time: 0.12, Decay time: 0.13, and Counts: 0.33, while that for the RMS feature is 0.0028. Therefore, the proposed PLIs are more sensitive to the leaks than the RMS feature. Thus, it can be concluded that the proposed PLIs not only have good leak sensing capability but also perform better than the previously used features, such as mean, variance, RMS, and kurtosis.

Overall, the proposed method can detect leaks without any prior leak information and is easy to implement. These qualities make the proposed method attractive for leak detection in the industries. In the future, the proposed method can be applied to AE signals along with stochastic resonance [29,30].

## 6. Conclusions

This paper proposed a leak detection approach using an acoustic emission technology. The proposed technique first extracts the AEE features from the AE signal. For this purpose, a sliding window was used with an adaptive threshold so that the properties of both burst- and continuous-type emissions can be retained. The proposed procedure uses the extracted AE signal features and two-sample KS test for building a leak indicator. The advantage of using AEE features is that they are an inherent property of the AE signals and help to capture the leak information more accurately than traditional statistical features. These individual AEE features form distributions that are different for the normal and leak conditions. The tested AEE feature distributions are then distinguished from the normal conditions by applying the two-sample KS test, which primarily measures the separation between the respective ECDFs and yields the leak indicator. The two-sample KS test offers an advantage that it is insensitive to extreme values in the distributions occurring because of outliers. The proposed approach was tested on experimental datasets collected from an industrial pipeline, and the results verified that the obtained PLIs remarkably predicted the leak conditions. In addition, the advocated PLIs outperformed the traditional features, mean, variance, RMS, and kurtosis in terms of leak recognition ability and leak sensitivity. Future work will study other well-known statistical tests, such as the T-square test and F-test, for condition monitoring of pipelines.

## Data Availability

The data is available upon the request.
